# Opportunities and challenges for hepatitis B cure in people living with HIV and hepatitis B virus

**DOI:** 10.1002/jia2.70015

**Published:** 2025-07-25

**Authors:** Kasha P. Singh, Jennifer Audsley, Wei Zhao, Sharon R. Lewin

**Affiliations:** ^1^ Victorian Infectious Diseases Service Royal Melbourne Hospital at the Peter Doherty Institute for Infection and Immunity Melbourne Victoria Australia; ^2^ Department of Infectious Diseases The University of Melbourne at The Peter Doherty Institute for Infection and Immunity Melbourne Victoria Australia; ^3^ Department of Infectious Diseases Alfred Hospital and Monash University Melbourne Victoria Australia

1

Despite effective antiviral treatment, hepatitis B virus (HBV) is the leading cause of cirrhosis and liver cancer globally, with over 250 million people living with chronic hepatitis B. Antiviral treatment for people with chronic hepatitis B is usually with just a single tablet a day and, for most people, continues lifelong [1]. Therefore, similar to HIV, there is high interest in developing a cure for chronic hepatitis B [2]. Of the 37 million people living with HIV (PWH), 7% are also living with chronic hepatitis B [3]. People living with HIV and HBV co‐infection present both challenges and opportunities to advance the field of HBV cure.

Chronic hepatitis B is defined as persistence of hepatitis B surface antigen (HBsAg) for at least 6 months. The natural history of chronic hepatitis B is characterized initially by high levels of HBV DNA in blood and a normal alanine aminotransferase (ALT), followed by intrahepatic inflammation with increased ALT, which can then progress to fibrosis, cirrhosis and hepatocellular carcinoma [4]. However, in contrast to HIV, a small percentage of people with chronic hepatitis B can lose HBsAg either spontaneously or following antiviral therapy [4]. The loss of HBsAg is associated with markedly reduced risk of liver disease and hepatocellular carcinoma [5], and is, therefore, considered a functional cure.

Multiple strategies are being developed to increase HBsAg loss. These include strategies to better block HBV replication, suppress production of HBsAg (which is immunosuppressive) or enhance HBV‐specific immunity (reviewed in [4, 6]) (Table 1). Some strategies being developed for HBV cure are also being investigated for HIV cure [10]. Examples include immune modulation with agents such as anti‐programmed death‐1 and toll‐like receptor agonists [10]. However, unfortunately, most people living with HIV and HBV co‐infection are excluded from both HIV and HBV cure clinical trials.

**Table 1:** Strategies for the cure of HBV (reviewed in [4, 6, 7])

Strategy	Target	Mechanism	Class and agents /examples and phase of development
Anti‐viral	HBV replication	Modulate HBV capsid assembly leading to aberrant/empty capsids, decreasing pre‐genomic RNA packaging, HBV protein production and HBV DNA replication	Capsid assembly modulators, for example JNJ‐6379 (Phase 2b)
		Interfere with HBV RNA, thereby inhibiting translation, HBV DNA replication and viral protein production (often GalNc^a^‐conjugated to target liver cells)	*Small interfering RNA* (siRNA): for example VIR‐2218 (Phase 2), JNJ‐3989 (Phase 2b) *Antisense oligonucleotides*: for example bepirovirsen (Phase 2)
		Block HBV entry into hepatocytes via the sodium taurocholate co‐transporting polypeptide receptor	Entry inhibitors, for example Buleviritide (Phase 3 for HDV)
	CccDNA [7]	Strategies such as inhibition of rcDNA to cccDNA conversion, epigenetic silencing, interference with repair factors, induction of apoptosis of infected cells and DNA cleavage or gene editing are all being explored to target HBV cccDNA	Agents in pre‐clinical development stage only [8].
	HBsAg	Block assembly and release from hepatocytes, potentially leading to enhanced HBV specific‐immune response	Nucleic acid polymers, for example REP‐2139, REP‐2165 (Phase 2)
			Neutralizing monoclonal antibody, for example VIR‐3434 (Phase 1)
Restoration and/or boosting of immune responses	Innate immunity	Toll‐like receptor activation	TLR‐7 agonist, for example ruzolotolimod (Phase 2) TLR‐8 agonist, for example selgantolimod (Phase 2)
	HBV‐specific adaptive immune response	Therapeutic vaccination to augment immune responses	Chimpanzee adenoviral and modified vaccinia Ankara viral vectors ChAdOx1‐HBV/MVA‐HBV [9] (Phase 2)
		Remove immune blockade, thereby reversing immune exhaustion to restore HBV‐specific T‐cell responses	Blockers of negative immunoregulatory pathways (checkpoint inhibitors), for example programmed cell death protein 1 (PD‐1), for example Nivolumab, Envafolimab (ASC22) (both Phase 2); and cytotoxic T lymphocyte‐associated protein 4 (CTLA‐4)
	Pegylated IFN‐α^b^	Enhance anti‐HBV immunity; direct antiviral effects inhibiting transcription of pgRNA and subgenomic RNA from cccDNA; activates lymphotoxin‐β receptor [4]	Pegylated IFN‐α is currently indicated for the treatment of chronic hepatitis B in some circumstances [1]. It is also being added to novel therapeutic interventions for HBV in clinical trials [4].

Abbreviations: APOBEC, Apolipoprotein B mRNA Editing Catalytic Polypeptide‐like; cccDNA, covalently closed circular DNA; GalNAc, N‐Acetylgalactosamine; HBsAg, hepatitis B surface antigen; HBV, hepatitis B virus; HDV, hepatitis D virus; IFN, interferon; siRNA, small‐interfering RNA.

^a^ GalNAc (N‐Acetylgalactosamine) selectively targets liver cells by binding to the asialoglycoprotein (ASGPR) receptor on hepatocytes.

^b^ Pegylated interferon‐α has multiple effects on HBV both directly antiviral and immune enhancing of both the adaptive immune responses.

Studies from the early 1990s showed very high liver‐related mortality among people living with HIV and HBV co‐infection compared to people with HBV mono‐infection, especially among those with low CD4+ T‐cell counts [11]. HBV‐active antiretroviral therapy (ART) that contains tenofovir (or tenofovir alafenamide), lamivudine (or emtricitabine) or both, suppresses replication of both HIV and HBV and improves health and life expectancy for people living with co‐infection. Interestingly, initiation of HBV‐active ART results in high rates of HBsAg loss with a prevalence of up to 20% in the first 2 years of treatment [12]. This is in contrast to HBsAg loss of only 1% per year following initiation of nucleo(s/t)ide reverse transcriptase inhibitors in HBV mono‐infection [13]. Therefore, understanding HBsAg loss in people living with HIV and HBV co‐infection could provide important insights into strategies to achieve an HBV cure.

The drivers of the high frequency of HBsAg loss in people living with co‐infection are currently unclear. It is important to note that in people living with co‐infection, everyone is treated for HBV at the time of HIV diagnosis, regardless of the phase of HBV infection. This is in contrast to people with HBV mono‐infection, where the criteria for initiation of antiviral therapy is the phase of HBV infection, namely the presence of liver damage as evidenced by an elevated ALT [14]. It is possible that antiviral treatment in the absence of liver inflammation may somehow result in a greater chance of HBsAg loss, although similar findings were not seen in a recent study of treatment initiation with tenofovir in the absence of liver inflammation in HBV mono‐infection [14]. Another possible explanation for high HBsAg loss in people living with co‐infection is immune dysregulation following initiation of ART. In people living with co‐infection and low CD4 counts, HBV‐related immune restoration disease and hepatic flare can occur following initiation of ART [15]. But even in the absence of immune restoration disease, elevation of specific cytokines or chemokines following ART initiation could favour enhanced HBV‐specific immunity. We recently showed in a large prospective cohort of people living with co‐infection who live in Thailand that HBsAg loss was associated with higher ALT, younger age, lower liver stiffness, lower baseline HBsAg and a tenofovir alafenamide‐containing regimen [16].

Similar to chronic hepatitis B, there is high interest in an HIV cure for PWH on lifelong ART. However, given the unknown effects of some HIV cure interventions on HBV and the increasing need to undergo a short period of stopping ART in HIV cure trials, people living with HIV and HBV co‐infection are generally excluded from HIV cure trials. Furthermore, the impact of HBV on the HIV reservoir is largely understudied. In another separate cohort of people living with co‐infection on ART in Thailand, we found that HIV DNA persists in the liver but is transcriptionally silent [17]. Further work understanding transcriptional control of HIV in the liver could provide new insights into HIV latency and the effect of the host cell on viral transcription, such as in the hepatocyte or in HIV‐infected T cells that traffic through the liver. Increased immune activation in people living with HIV and HBV co‐infection may alter the establishment, maintenance and potentially reversal of HIV latency. However, to date, few studies have specifically addressed whether the size and composition of the latent HIV reservoir differ between PWH and people living with co‐infection on long‐term suppressive ART.

Despite the multiple interventional studies currently underway for the cure of either chronic hepatitis B or HIV, we are aware of only one interventional study for HBV cure in people living with HIV and HBV co‐infection [18]. This study is investigating the role of selgantolimod (a TLR8 agonist) on HBsAg loss in people living with co‐infection, given prior promising reports of selgantolimod in HBV mono‐infection [19]. TLR8 agonists can also reverse HIV latency as shown in in vitro studies [20], and this study also represents an exciting opportunity to understand the effects of selgantolimod on the HIV reservoir.

The higher rates of HBsAg loss in people living with HIV and HBV co‐infection provide a unique opportunity to understand the mechanism of HBsAg loss. Given the significant number of people living with co‐infection, it is vital to examine new HBV cure therapeutics in them. We propose that people living with both HIV and hepatitis B should be a priority population for such studies and not excluded from. People living with HIV and HBV co‐infection should not be left behind in the journey towards an HBV or an HIV cure.

## COMPETING INTERESTS

SRL receives research support from the National Health and Medical Research Council of Australia (NHMRC) and the Victorian Government Department of Health and the National Institutes of Health. She has received consultancy fees from Abivax, Geovax, ViiV, Tetralogic, Vaxxinity and Esfam; and Honoraria from Gilead Sciences, Abbvie and Merck. All other authors have no potential competing interests to declare.

## AUTHORS’ CONTRIBUTIONS

KPS, JA, WZ and SRL contributed ideas, drafted and reviewed the manuscript, and approved the final article.
